# HIV-1 Tat: Molecular Switch in Viral Persistence and Emerging Technologies for Functional Cure

**DOI:** 10.3390/ijms26136311

**Published:** 2025-06-30

**Authors:** Kaixin Yu, Hanxin Liu, Ting Pan

**Affiliations:** Shenzhen Key Laboratory for Systems Medicine in Inflammatory Diseases, School of Medicine, Shenzhen Campus of Sun Yat-sen University, Sun Yat-sen University, Shenzhen 518107, China; yukx@mail2.sysu.edu.cn (K.Y.); liuhx66@mail2.sysu.edu.cn (H.L.)

**Keywords:** HIV-1 Tat, molecular switch, viral latency, CRISPR-dCas9, lipid nanoparticles, epigenetic engineering, functional cure

## Abstract

HIV-1 Tat acts as a central molecular switch governing the transition between viral latency and active replication, making it a pivotal target for HIV-1 functional cure strategies. By binding to the viral long terminal repeat (LTR) and hijacking host transcriptional machinery, Tat dynamically regulates RNA polymerase II processivity to alter viral transcription states. Recent studies reveal its context-dependent variability: while Tat recruits chromatin modifiers and scaffolds non-coding RNAs to stabilize epigenetic silencing in latently infected cells, it also triggers rapid transcriptional amplification upon cellular activation. This review systematically analyzes the bistable regulatory mechanism of Tat and investigates advanced technologies for reprogramming this switch to eliminateviral reservoirs and achieve functional cures. Conventional approaches targeting Tat are limited by compensatory viral evolution and poor bioavailability. Next-generation interventions will employ precision-engineered tools, such as AI-optimized small molecules blocking Tat-P-TEFb interfaces and CRISPR-dCas9/Tat chimeric systems, for locus-specific LTR silencing or reactivation (“block and lock” or “shock and kill”). Advanced delivery platforms, including brain-penetrant lipid nanoparticles (LNPs), enable the targeted delivery of Tat-editing mRNA or base editors to microglial reservoirs. Single-cell multiomics elucidates Tat-mediated clonal heterogeneity, identifying “switchable” subpopulations for timed interventions. By integrating systems-level Tat interactomics, epigenetic engineering, and spatiotemporally controlled delivery, this review proposes a roadmap to disrupt HIV-1 persistence by hijacking the Tat switch, ultimately bridging mechanistic insights to clinical applications.

## 1. Introduction

Since HIV-1’s identification as the causative agent of AIDS in 1983 [1], it has claimed over 42.3 million lives worldwide, with approximately 39.9 million people currently living with the virus [UNAIDS 2025]. While antiretroviral therapy (ART) has achieved remarkable success in effectively suppressing viral replication to undetectable levels, achieving a functional cure remains a challenge. This challenge stemsprimarily from viral latency, where integrated proviruses remain transcriptionally silent in long-lived CD4^+^ T cells and various tissue reservoirs, such as brain microglia and gut-associated lymphoid tissue [2,3,4,5]. These reservoirs, established within days of infection, persist indefinitely through clonal expansion and epigenetic silencing, enabling viral rebound upon ART cessation. Although lifelong ART is lifesaving, it fails to eradicate latent proviruses and introduces complications including drug resistance, chronic inflammation, and associated comorbidities [6,7,8]. This underscores the urgent need to develop sterilizing or functional cure strategies.

Central to viral persistence lies HIV-1 Tat, a master regulatory protein that functions as a bistable molecular switch governing the transition between latent and active viral states [9]. In activated CD4^+^ T cells, Tat recruits the host super-elongation complex (SEC; e.g., CDK9/P-TEFb) to phosphorylate RNA polymerase II, enabling transcriptional elongation and explosive viral replication [10]. Conversely, in quiescent reservoirs, Tat engages epigenetic silencers (e.g., Polycomb repressor Enhancer of zeste homolog 2, EZH2 [11]) and scaffolds non-coding RNAs (e.g., NRON/lncRNA [12]) to stabilize latency by enforcing LTR-driven transcriptional dormancy.Endogenously expressed Tat in latently infected T cells further maintains viral persistence by directly disrupting host transcriptional programs, exemplified by its suppression of IL-2 and exclusion of RNA polymerase II from the IL-2 promoter [13]. This dynamic equilibrium not only sustains viral persistence but also makes Tat a key target for treatment. Beyond transcription, Tat amplifies pathogenesis through neurotoxic signaling [14,15], immune evasion via MHC-II downregulation [16], and fostering viral mutagenesis through error-prone reverse transcription. These findings collectively underscore the necessity for precise and targeted therapeutic interventions.

Recently, the remarkable advancements in technology have been reshaping the development of cure strategies specifically targeting Tat, thereby facilitating significant progress toward meaningful breakthroughs. Innovations in nanoparticle delivery systems, machine learning platforms, and paradigm-shifting approaches leverage Tat bistable for silencing (“block and lock”) or activation (“shock and kill” [17,18,19]), effectively repurposing the viral switch against itself.

In this review, we start by exploring the role of Tat in managing HIV-1 latent reservoirs, focusing on its bistable functionality that controls latency and reactivation mechanisms. Subsequently, we critically evaluate emerging technologies, assessing their potential to overcome reservoirs by targeting Tat. We propose an integrative strategy that combines epigenetic precision, spatiotemporal delivery control, and immune checkpoint modulation to repurpose Tat from a driver of viral persistence into a therapeutic vulnerability. By bridging molecular virology with synthetic biology, this work aims to provide a paradigm shift from viral suppression to definitive cure.

## 2. The Role of Tat in the HIV-1 Viral Persistence

HIV-1 can infect a variety of cell types, including memory CD4^+^ T cells, macrophages, and dendritic cells, to establish latent reservoirs [20,21,22]. Among these, the integration of HIV-1 DNA into resting memory CD4^+^ T cells represents the most predominant HIV-1 latent reservoir due to the long lifespan of memory CD4^+^ T cells and their capacity to evade immune surveillance. Reactivating dormant HIV-1 is a pivotal step in the “shock and kill” strategy, and the levels of Tat protein play a critical role in governing the equilibrium between HIV-1 latency and reactivation. The Tat facilitates both the initiation and elongation of HIV-1 mRNA transcription, and its expression is dynamically regulated through transcriptional, post-translational, and spatial mechanisms. These regulatory processes offer multiple therapeutic targets for modulating viral persistence.

### 2.1. Mechanisms of Latent Reservoir Establishment and Persistence

HIV-1 establishes viral reservoirs through three canonical pathways: (1) Transitional latency: Activated—a CD4^+^ T lymphocytes that initially support productive infection revert to a quiescent state post-integration, harboring transcriptionally silent proviruses [23]. (2) Direct infection of resting CD4^+^ T cells—HIV-1 exploits low-energy entry pathways to directly infect resting memory CD4^+^ T cells, thereby bypassing activation-dependent replication checkpoints [24,25]. (3) Integration site bias: —proviral integration preferentially occurs in genomic regions with low gene density (e.g., heterochromatic regions and gene-poor chromosomal areas) or in antisense orientations relative to host transcription units, particularly in naïve and effector CD4^+^ T cells [24,25,26,27]. Notably, while activated effector CD4^+^ T cells predominantly sustain lytic infection, the direct infection of central and transitional memory CD4^+^ T cells favors the establishment of latency [26].

Memory CD4^+^ T cells exhibit prolonged survival and self-renewal capabilities, which are essential for the long-term maintenance of viral reservoirs. The persistence of these reservoirs is driven by complex interactions between molecular and cellular dynamics. Clonal proliferation serves as the primary mechanism. Large-scale clonal expansion of infected cells harboring intact or defective proviruses significantly contributes to reservoir persistence. Notably, ≥50% of latent CD4^+^ T cells undergo clonal expansion [8], driven by mechanisms such as homeostatic proliferation, antigen-driven proliferation, and integration site-mediated proliferation [28,29]. Proviruses that lack protein expression but retain splicing capability may further enhance clonal expansion [30]. Concurrently, transcriptional interference represents another critical factor. HIV-1 transcriptional interference is influenced by functional differences and theintegration orientation of LTRs. Typically, the 5′LTR initiates transcription, while the 3′LTR terminates transcription due to RNAPII blockage or reduced transcription factor binding. When viral LTR orientation aligns with the host gene direction, RNAPII generates nonfunctional transcripts; in the opposite orientation, it produces long hybrid mRNAs. HIV-1 transcription resumes under conditions where host genes are silenced [2]. Furthermore, epigenetic modifications, including transcriptional regulation and post-translational histone modifications (e.g., acetylation and methylation), modulate HIV-1 transcription post-integration, often suppressing proviral transcription. These effects are reinforced by epigenetic barriers, such as Polycomb-repressive complex 2 (PRC2)-mediated H3K27me3 marks, LTR CpG hypermethylation, and Lens epithelium-derived growth factor (LEDGF/p75)-mediated tethering of proviruses to transcriptionally inert nuclear regions [11,27,31,32].

Further complexity arises from microenvironmental crosstalk and the transcriptional pausing machinery. Cell-to-cell contact between infected and uninfected cells, as well as cytokines secreted by relevant cells, may influence the sensitivity and infection status of resting CD4^+^ T cells to HIV-1 infection. In co-culture experiments with endothelial cells or macrophages, resting CD4^+^ T cells exhibit enhanced susceptibility to HIV-1 infection [33]. An in vivo study demonstrated that CD8^+^ T lymphocytes inhibit the reactivation of the HIV-1 latent reservoir by the latency-reversing agent N-803 [34]. Co-cultured cells can affect resting CD4^+^ T cells via two mechanisms: cell-to-cell viral transmission [33] or the release of acellular viral particles by infected cells [35].

At the transcriptional level, negative elongation factor (NELF) and DRB sensitivity-inducing factor (DSIF) maintain HIV-1 latency by modulating RNAP II activity [27]. P-TEFb, composed of a cyclin-dependent kinase (CDK9) and its regulatory partner Cyclin T1, plays a critical role in regulating Pol II activity. A reduction in free P-TEFb blocks HIV-1 transcription, contributing to the establishment of a latent reservoir. Additional transcription factors, such as CycK/CDK13 and Cyclin/CDK11, are also involved in HIV-1 transcription; for instance, CDK11 influences the cleavage and polyadenylation of HIV-1 transcripts [2]. LEDGF/p75, a chromatin-associated factor, promotes HIV-1 latency by recruiting the Pol II-associated factor 1 (PAF1) complex to maintain Pol II pausing at the LTR region [32]. Long non-coding RNAs (lncRNAs) contribute to latent maintenance through mechanisms such as inducing autophagy [36], modulating HIV-1 transcription [37,38], and regulating post-transcriptional stages of HIV-1 [39]. Additionally, microRNAs promote latent maintenance by targeting HIV-1 RNA [40,41,42], transcription factors [43,44,45], and host dependency factors [46,47].

This multilayered regulation—spanning clonal dynamics, chromatin topology, and immune modulation—establishes a robust equilibrium that resets the Tat-mediated molecular switch toward latency, underscoring the need for combinatorial strategies to disrupt reservoir resilience.

### 2.2. Tat as a Reactivation Driver

The reactivation of HIV-1 latent reservoirs—a key determinant of viral rebound—depends on both stochastic viral transcription and Tat-mediated regulation. The levels of Tat protein are closely associated with viral latency, serving as a bistable molecular switch that dictates the transition between transcriptional silence and explosive reactivation. In the absence of Tat, HIV-1 transcription is halted at the promoter-proximal paused state, yielding only short abortive transcripts that reinforce latency via RNA-mediated chromatin compaction. Conversely, the presence of Tat initiates a nonlinear transcriptional amplification cascade, increasing the production of full-length RNA transcripts (>59 nt) by over 100-fold through positive feedback loops [48]. As the viral transactivator, Tat is crucial in reversing latency through its interactions with host transcriptional machinery, epigenetic modifiers, and signaling pathways. These multifaceted interactions enable Tat to reprogram the host epigenome, thereby creating an environment conducive to viral replication.

The primary role of Tat lies in overcoming transcriptional pausing by hijacking the P-TEFb, a rate-limiting host cofactor. Through its arginine-rich motif (ARM), Tat displaces HEXIM1 from the inhibitory 7SK snRNP complex, consequently liberating P-TEFb (CycT1/CDK9) to form a ternary complex with Tat and SEC. This complex binds to the TAR RNA stem-loop with nanomolar affinity, positioning CDK9 to phosphorylate two critical targets: Spt5 and RNAPII. Spt5, a subunit of NELF, upon phosphorylation, disrupts interactions between NELF and RNA, thereby eliminating transcriptional barriers. Meanwhile, the phosphorylation of RNAPII at Ser2 residues within its C-terminal domain (CTD) facilitates transcriptional elongation by recruiting mRNA capping and splicing factors [2].

Tat further enhances transcription initiation by recruiting components of the pre-initiation complex (PIC), such as Mediator and TATA-binding protein, along with histone acetyltransferases (p300/CEBP, PCAF, and hGCN5) to the viral LTR. p300 acetylates histones H3K27 and H4K16, thereby erasing repressive chromatin marks and improving promoter accessibility [2]. Recent single-molecule imaging studies highlight a concentration-dependent duality in Tat’s function: while sub-stoichiometric levels of Tat promote latency through the generation of abortive short transcripts, elevated Tat expression in HIV-1C strains can trigger a negative feedback mechanism through competitive binding to TAR. This process recruits Histone deacetylase 1/Bromodomain containing 4 (HDAC1/BRD4) complexes, thereby silencing LTR activity. This exemplifies a bistable behavior that permits context-dependent viral decision-making [49].

Beyond transcriptional elongation, Tat modulates latency through four synergistic axes: (1) Epigenetic Rewiring—Tat inducesthe ROS/AKT-mediated phosphorylation of EZH2 at Ser21, which weakens the interaction between EZH2 and other PRC2 subunits, resulting in a genome-wide reduction in H3K27me3 levels and inhibiting H3K27 methyltransferase activity [11]. (2) miRNA Crosstalk—Tat downregulates miR-28 and miR-125b (which directly target 3’UTR regions) through NF-κB suppression while upregulating miR-34a to degrade mRNAs of host restriction factors (e.g., SIRT1) via Ago2-mediated silencing [50,51]. (3) Post-Translational Partnerships—Tat collaborates with Viral Protein R (Vpr) to hijack the CRL4-DCAF1 E3 ligase, leading to the ubiquitination and degradation of the latency factor CTIP2. Concurrently, Tat-Nef complexes stabilize STAT3, activating pro-viral cytokines (e.g., IL-6 and IL-10), while Tat interacts with nucleocapsid or Rev promoteand s the ubiquitination and proteasomal turnover of Tat [52,53,54]. (4) Ubiquitination Circuits—Tat recruits USP11 to deubiquitinate and stabilize SMYD family member 5 (SMYD5), a histone methyltransferase that deposits H4K20me1 at the LTR. SMYD5 also recruits BRD4, generating a feedforward loop that sustains Tat-P-TEFb activity [55].

Collectively, these mechanisms establish Tat as a central player in the viral reactivation process. According to Table 1, it integrates multiple regulatory levels—including concentration-dependent bistability, transcriptional regulation, and auxiliary mechanisms—to maintain viral activity. Targeting these pathways, particularly the interaction between Tat and P-TEFb along with their associated epigenetic cofactors, represents a promising strategy for destabilizing the latent viral reservoir.

### 2.3. The Regulation of Tat: A Multilayered Control Network Governing Viral Fate

The functionality and expression levels of Tat serve as a central rheostat that regulates the transition of HIV-1 between latency and reactivation. Subthreshold Tat transcription, such as defective LTR promoters or mutations in its activation domain, locks proviruses into deep latency by aborting transcriptional initiation [56,57]. Conversely, the depletion of Tat via RNA interference reduces reactivation efficiency by more than 80%, highlighting its role as a molecular amplifier [58]. This dynamic equilibrium is regulated by a tripartite hierarchical system spanning translational control, post-translational modifications, and subnuclear trafficking, with each layer finely tuning the activity of Tat to adapt to host cell states (Table 2).

The Tat-IRES modulator (TIM-TAM), a conserved 58-nucleotide RNA stem-loop located within the Tat ORF, functions as a riboswitch-like element that toggles between cap-dependent and cap-independent translation modes. Structural studies reveal its dual functionality. During the initial infection of activated CD4^+^ T cells, TIM-TAM adopts an open conformation that exposes an internal ribosome entry site (IRES), allowing for cap-independent Tat synthesis and bypassing mTOR-mediated translational checkpoints. The rapid accumulation of Tat consequently drives the initiation of viral replication while inducing lymphocyte proliferation through ERK/MAPK crosstalk [59,60]. In resting memory T cells, TIM-TAM folds into a closed structure that sequesters the Tat initiation codon (AUG), sterically hindering the scanning of 43S ribosomes. Single-molecule ribosome profiling indicates that this conformation reduces cap-dependent Tat translation by sixfold-, favoring the establishment of latency [61]. The CRISPR-directed disruption of TIM-TAM (e.g., G26C mutation) collapses this balance, locking 92% of proviruses in latency by destabilizing the bistability of Tat expression [60].

The transactivation potency of Tat is also shaped by a kinetic competition between activating modifications and destabilizing signals. Protein arginine methyltransferase(PRMT) family (PRMT2 and PRMT6) catalyze the asymmetric dimethylation of Tat’s R52/R53 residues. This modification (i) disrupts Tat-TAR RNA binding by neutralizing the positive charge of arginine (K_d_ increases from 2 nM to >200 nM) and (ii) blocks CycT1 interaction, precluding the recruitment of P-TEFb to the LTR [25]. The nucleolar protein Nuleolar Protein2/Nucleolin (NOP2/NCL) executes a dual attack: (i) it directs m^5^C methylation of TAR RNA at C5/C34, impairing Tat-TAR recognition, and (ii) competitively binds to TAR with tenfold- higher affinity than Tat (K_d_ = 0.3 nM vs. 3 nM), sequestering viral RNA into nucleolar detention centers [58]. UHRF1, an E3 ubiquitin ligase, tags Tat’s lysine 50/51 residues with K48-linked polyubiquitin chains, targeting Tat for 26S proteasomal degradation. The pharmacological inhibition of UHRF1 increases Tat’s half-life from 2 h to over 8 h, leading to the reactivation of 65% of latent proviruses in primary CD4^+^ T cells [62].

The functional output of Tat is still spatially regulated through competitive interactions with scaffold proteins. Nucleophosmin 1(NPM1) binds Tat’s basic domain via its oligomerization interface, shuttling Tat into nucleoli. Within this transcriptionally inert compartment, Tat is trapped in phase-separated NPM1 condensates, reducing its nuclear availability by 70% [25]. The SEC scaffold ALF transcription elongation Factor4 (AFF4) competes with NPM1 for Tat binding, redirecting Tat to nuclear speckles enriched with RNAPII and P-TEFb. CRISPR knockout of AFF4 shifts Tat’s nucleolar: nucleoplasmic ratio from 1:3 to 4:1, significantly reducing transcriptional elongation efficiency [25]. Furthermore, the C-terminal domain of Tat drives liquid–liquid phase separation (LLPS) with SEC components. Mutations disrupting LLPS abolish Tat-mediated transcriptional clusters, leading to a 90% reduction in viral mRNA output [55].

This multilayered regulation establishes Tat as a kinetically gated switch: TIM-TAM controls Tat synthesis thresholds, post-translational modifications modulate its functional half-life, and scaffold competition determines spatial efficacy. Single-cell RNA-seq analysis reveals that fewer than 5% of latently infected cells spontaneously achieve the Tat concentration threshold (>200 molecules/cell) required to overcome these regulatory barriers, thereby explaining reservoir stochasticity [63]. Emerging strategies aim to perturb this balance: TIM-TAM-targeted antisense oligonucleotides (ASOs) lock the RNA switch in its closed state, enforcing latency. While TIM-TAM-targeted ASOs induce latency in vitro by stabilizing the closed RNA conformation, their in vivo delivery is challenged by factors such as renal clearance and nuclease degradation. PRMT6 inhibitors (e.g., EPZ020411) prevent Tat methylation, enhancing its transactivation potential for “shock and kill” approaches. NPM1 degraders (e.g., CX-5461) release nucleolar Tat pools, sensitizing reservoirs to latency reversal.

By elucidating the functional mechanisms of Tat, we can effectively reprogram the central control system of HIV-1, converting its survival strategy into a critical vulnerability.

**Table 2:** The regulation of Tat’s activity.

Modification Type	Enzymes/Effectors	Functional Outcome
Inhibitory	PRMT2 and PRMT6	Asymmetric arginine dimethylation attenuates Tat-P-TEFb binding [25].
	UHRF1	K48-linked ubiquitination targets Tat for proteasomal degradation [62].
Activating	p300/CEBP	Lysine acetylation enhances Tat’s nuclear localization and LTR binding [64].
	USP7	Deubiquitination stabilizes Tat, promoting transcriptional elongation [65].
Others	NOP2/NSUN1	A nucleolar RNA methyltransferase silences Tat by dual mechanisms: catalyzing TAR RNA m5C methylation and competitively blocking Tat-TAR interactions [58].
	NPM1	sequestersS Tat in the nucleolus, limiting its access to the LTR [25].
	AFF4	Recruits Tat to nuclear speckles, facilitating SEC assembly at transcriptionally actives [25].

## 3. Targeting Tat: Therapeutic Strategies

### 3.1. Barriers and Challenges: The Spatiotemporal Complexity and Reservoir Adaptability of Tat

The path to HIV-1 cure strategies is obstructed by the spatiotemporal complexity of Tat functionality and the adaptability of viral reservoirs. In the central nervous system, brain-adapted Tat isoforms exhibit dual pathological reprogramming: they evade latency-reversing agents through low-affinity TAR RNA binding (Kd ≈ 50 nM) while driving neuroinflammation via TLR4/NF-κB-mediated microglial activation and Matrix metalloproteinase-9-dependent(MMP-9-dependent) blood–brain barrier breakdown. Concurrently, these variants suppress astrocytic glutamate transporters (EAAT2) through miR-218-5p, elevating extracellular glutamate to neurotoxic levels (≥100 μM)—a mechanism directly correlating with HIV-associated neurocognitive disorders. Contrastingly, in lymphoid niches like T follicular helper cells and gut-associated lymphoid tissue, Tat hijacks chemokine signaling axes (CXCR4/CXCL12 and CCR5/CCL5) to synchronize viral replication with host activation cycles while upregulating immune checkpoints to create “immune-privileged” clones paradoxically susceptible to checkpoint inhibitor-induced reactivation. Adipose and genital reservoirs further complicate this landscape, where Tat co-opts PPARγ signaling to polarize tissue-resident macrophages into immunosuppressive M2 phenotypes, establishing IL-35/TGF-β-enriched sanctuaries resilient to conventional therapies.

The functionality of Tat is profoundly influenced by tissue-specific microenvironments and reservoir heterogeneity, creating dynamic challenges for cure strategies.

In the brain, Tat exhibits unique neurotoxic properties, promoting inflammation and blood–brain barrier dysfunction. HIV-1-infected microglia and astrocytes harbor Tat variants with reduced transcriptional activity but enhanced capacity to evade immune surveillance. These “brain-adapted” Tat isoforms (e.g., C31S mutant) resist reactivation by conventional latency-reversing agents (LRAs), contributing to persistent neurocognitive disorders. The Tat-mediated suppression of astrocytic glutamate transporters exacerbates neuronal excitotoxicity, linking viral persistence to neurodegeneration. In lymph nodes and gut-associated lymphoid tissue (GALT), Tat drives localized immune activation, fostering viral replication in CD4^+^ T follicular helper (Tfh) cells. Proviruses integrated into *CXCR4* or *CCR5* co-receptor genes exploit chemokine signaling to enhance clonal survival. Tat enhances PD-1 expression on infected Tfh cells, promoting immune evasion while paradoxically sensitizing cells to PD-1 blockade therapies. In genital and adipose tissues, Tat modulates tissue-resident macrophage polarization, creating niches for viral persistence through IL-10-mediated immunosuppression.

Latent reservoirs are not static entities but genetically mosaicked populations shaped by clonal selection pressures. Proviruses integrated into STAT5B or BTB domain and CNC homolog 2 (BACH2) exploit host super-enhancers for clonal expansion while maintaining transcriptional silence through BRD4/NuRD-mediated chromatin repression, whereas those antisense to Metastasis-Associated Lung Adenocarcinoma Transcript 1/Nuclear Enriched Abundant Transcript 1 (MALAT1/NEAT1) lncRNAs produce chimeric transcripts that mask Tat mRNA from innate sensors, enabling stochastic viral “blips”. Dominant-negative Tat mutants (K41A and R57Q) evolve under cytotoxic T lymphocyte pressure, reducing MHC-I antigen presentation by 80% through impaired TAP binding while competitively inhibiting wild-type Tat via TAR RNA squelching [66]. Even defective proviruses contribute to persistence—truncated Tat peptides oligomerize with functional counterparts, sequestering them in cytoplasmic aggresomes. This heterogeneity is compounded by epigenetic diversification: single-cell analyses reveal that <10% of clones possess Tat-responsive bivalent chromatin domains (H3K4me3/H3K27me3), necessitating precision epigenome editing to prime latent proviruses for targeted reactivation.

Therapeutic targeting of Tat faces a paradoxical trade-off: its centrality in viral reactivation makes it indispensable, yet its evolutionary plasticity fosters resistance. Brain-penetrant Tat inhibitors must navigate efflux pumps (P-gp/BCRP) and avoid off-target N-methyl-D-aspartate (NMDA) receptor blockade, while gut-targeted Proteolysis Targeting Chimeras (PROTAC) degraders risk neurotoxic Tat fragment accumulation. Combinatorial approaches—pairing dCas9-SunTag/Tet1-mediated LTR demethylation with cytotoxic T lymphocyte (CTL)-redirecting bispecific antibodies—show promise in overcoming clonal evasion but require nanoscale delivery systems to penetrate anatomical sanctuaries. Ultimately, dismantling HIV persistence demands systems-level strategies that map clonal architectures in 4D (space-time), override tissue-specific Tat adaptations, and preempt evolutionary escape routes—a multidisciplinary frontier spanning synthetic biology, neuroimmunology, and computational virology.

### 3.2. Conventional Tat-Targeted Strategies

**Figure 1:**
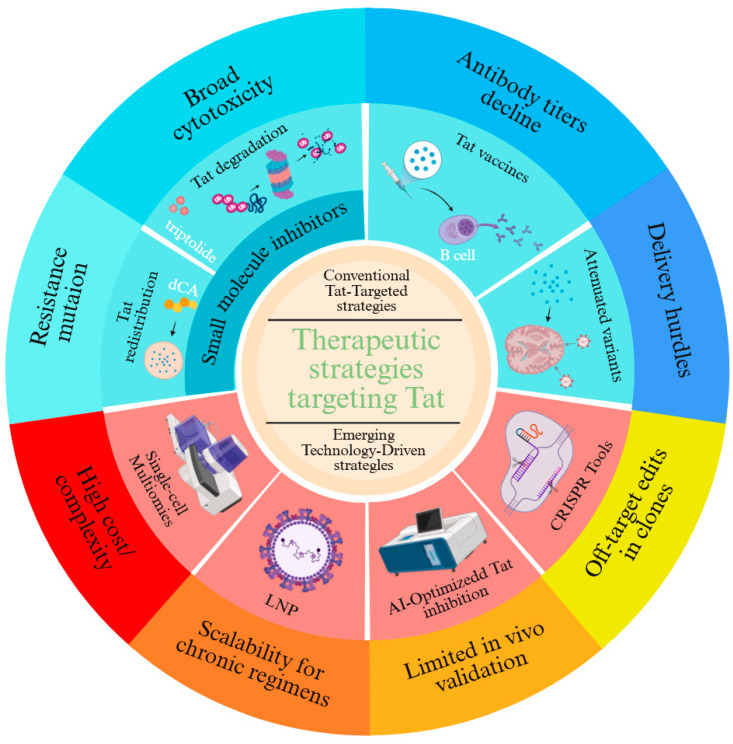
Therapeutic Strategies Targeting Tat.

Tat, the viral transactivator essential for HIV transcriptional elongation, has emerged as a linchpin for therapeutic strategies aimed at achieving a functional cure. Conventional approaches focus on exploiting Tat’s role in latency reversal or enforcing its silencing to suppress viral rebound, yet face challenges rooted in viral adaptability and tissue-specific barriers (Figure 1).

#### 3.2.1. The Mechanism of Conventional Approaches

Small-molecule Tat inhibitors, such as didehydro-cortistatin A (dCA), bind the arginine-rich motif (ARM) of Tat to disrupt its interaction with P-TEFb and TAR RNA, redistributing Tat from transcriptionally inert nucleoli to the nucleoplasm [67,68]. Preclinical studies demonstrate that dCA reduces residual viral transcription by >90% in humanized mouse models, delaying viral rebound for up to 27 days post-ART cessation. However, resistance rapidly evolves through LTR mutations (e.g., TAR bulge deletions) and compensatory accessory gene adaptations (Neferin-mediated immune evasion and Vpr-driven T cell activation). Similarly, triptolide—a plant-derived diterpenoid—promotes Tat ubiquitination and proteasomal degradation but exhibits broad cytotoxicity by indiscriminately inhibiting RNAPII [69]. To address specificity, second-generation compounds like Q308 combine Tat degradation (via cereblon E3 ligase recruitment) with BRD4 displacement, selectively inducing apoptosis in infected cells through c-Myc suppression [70].

Vaccine strategies aim to harness Tat’s immunogenicity for host-mediated viral control. Phase II trials of recombinant Tat vaccines (Tat-BH10 and Tat-Oyi) demonstrated cross-clade antibody responses in 70–90% of recipients, correlating with a 0.5-log reduction in proviral DNA and delayed rebound (median 8.6 weeks post-ART). However, antibody titers decline by 33% within 12 months, reflecting waning germinal center activity [63,71]. Next-generation platforms, such as nucleoside-modified mRNA-LNP encoding conserved Tat epitopes, enhance CD4^+^ T follicular helper cell priming and durability by mimicking natural antigen presentation [72]. Combinatorial approaches pairing Tat vaccines with Env gp140 immunogens further broaden immune recognition, neutralizing 68% of tier-2 viruses in nonhuman primates.

Gene-editing and RNA interference strategies face delivery hurdles despite mechanistic precision. Attenuated Tat variants (e.g., tat-R5M4) retain 40% transcriptional activity while reducing neurotoxicity by ablating chemokine mimicry, yet in vivo delivery via lentiviral vectors risks insertional mutagenesis. CRISPR-dCas9 systems fused with Krüppel-associated box (KRAB) domains achieve locus-specific LTR silencing but exhibit off-target editing in 12% of genomic sites. RNAi approaches, including lipid nanoparticle-encapsulated siRNA targeting tat exon 2, suppress viral replication by 85% in lymphoid tissue but fail to penetrate CNS reservoirs due to blood–brain barrier efflux. Emerging solutions include engineered transcription activator-lkei effector nucleases (TALENs) with enhanced nuclear localization signals and AAV9-based vectors for neuronal targeting, though immunogenicity and clonal escape remain persistent challenges.

Collectively, these conventional strategies underscore Tat’s dual role as a therapeutic Achilles’ heel and a moving target, necessitating combinatorial approaches that integrate precision targeting with adaptive immune modulation to outmaneuver viral evolution.

#### 3.2.2. The Limitations of Conventional Approaches

The quest for an HIV functional cure has crystallized around two complementary paradigms: the “block and lock” strategy, which enforces the transcriptional silencing of latent proviruses, and the “shock and kill” approach, designed to purge reservoirs through targeted reactivation. Both hinge on manipulating Tat—the viral transactivator that serves as HIV’s molecular switch—yet face intrinsic limitations rooted in viral adaptability and biological complexity.

The “block and lock” strategy employs small molecules like didehydro-cortistatin A (dCA) to disrupt Tat’s interaction with the P-TEFb complex, epigenetically silencing the HIV promoter by maintaining repressive histone marks (H3K27me3) and DNA hypermethylation at the LTR [73]. While dCA reduces residual transcription by >80% in vitro, its efficacy is undermined by proviral heterogeneity: 30–40% of latent reservoirs exhibit Tat-independent basal transcription via host transcription factors (NF-κB/SP1), enabling viral escape. Conversely, “shock and kill” relies on latency-reversing agents (LRAs) to reactivate Tat-dependent transcription, yet even potent PKC agonists (e.g., bryostatin-1) achieve <5% reservoir activation in clinical trials, limited by Tat expression stochasticity and tissue-specific chromatin barriers [74]. Histone deacetylase inhibitors like romidepsin further illustrate this paradox—while globally increasing histone acetylation, they fail to dislodge Tat from nucleolar sequestration in CNS reservoirs, leaving 60–70% of brain-harbored proviruses untouched.

Persistent challenges stem from the anatomic and clonal stratification of reservoirs. Resting CD4^+^ T cells in lymphoid tissues maintain latency through Tat proteasomal degradation (UHRF1-mediated), whereas microglial reservoirs in the CNS retain Tat isoforms (e.g., C31S) resistant to LRAs due to impaired TAR RNA binding [66]. Furthermore, clonal expansion dynamics—driven by proviral integration near survival genes like *BACH2*—create self-renewing populations that evade immune detection through Tat mutation mosaicism (e.g., K41A reducing MHC-I presentation by 70%) [66]. Even when reactivated, <1% of reservoir cells express sufficient surface HIV antigens to trigger CTL responses, underscoring the need for precision adjuvants.

Emerging solutions aim to transcend these barriers through Tat-centric engineering. CRISPR-dCas9 systems fused with Krüppel-associated box (KRAB) domains achieve locus-specific LTR silencing (“block and lock”) by recruiting DNA methyltransferase 3A (DNMT3A) and histone deacetylase 4(HDAC4), reducing off-target effects tenfold- compared to small molecules [75]. For “shock and kill,” lipid nanoparticles (LNPs) deliver *tat* mRNA alongside bromodomain and extraterminal inhibitors (BETis), generating synchronized Tat pulses (>200 molecules/cell) that overcome chromatin repression in 90% of gut-associated reservoirs [72]. Combinatorial regimens pairing these tools with PD-1 blockade or bispecific antibodies (CD3xEnv) enhance infected cell clearance from 12% to 65% in humanized mouse models. However, Tat’s pleiotropic roles—activating neurotoxic cytokines (TNF-α, IL-6) in astrocytes while suppressing antiviral interferon-induced transmembrane protein3 (IFITM3) in macrophages—demand spatiotemporal control to avoid collateral damage.

Thus, while conventional strategies illuminate Tat’s centrality in HIV persistence, their limitations underscore a pivotal truth: durable cure requires systems-level interventions that resolve Tat’s duality—exploiting its transcriptional mastery while neutralizing its pathological offshoots—through synthetic biology, immune redirection, and artificial intelligence-driven design (Table 3).

### 3.3. Emerging Technology-Driven Strategies

**Figure 2:**
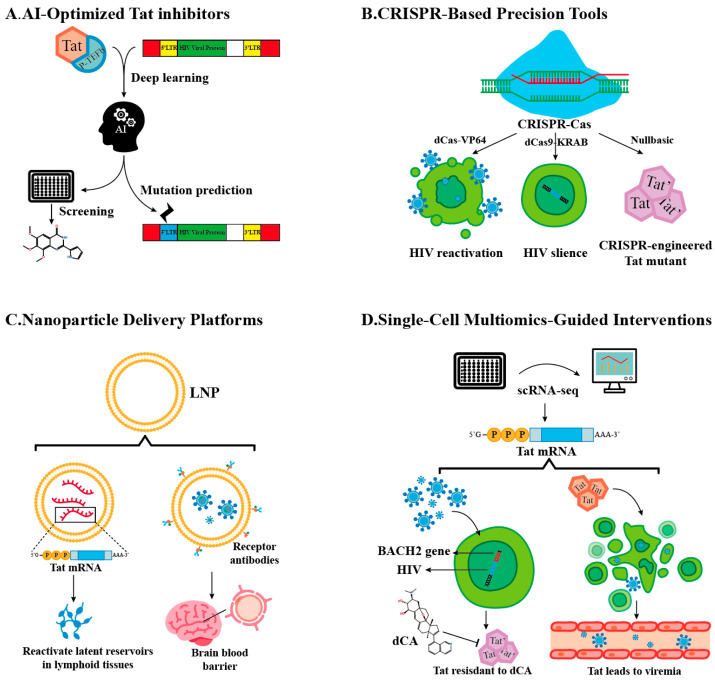
Technology-Driven Strategies.

The limitations of conventional approaches have spurred the development of innovative, technology-driven strategies that exploit Tat’s unique biology. Below, we highlight five transformative avenues reshaping Tat-targeted HIV cure research (Figure 2) along with the advantages and challenges of these avenues (Table 4).

#### 3.3.1. AI-Optimized Tat Inhibitors

Artificial intelligence (AI) is revolutionizing Tat inhibitor design by enabling atomic-level precision in targeting HIV-1’s master transcriptional regulator. Leveraging deep learning architectures trained on multi-modal datasets—including cryogenic Electron Microscopy (cryo-EM) structures of Tat-P-TEFb-TAR complexes, molecular dynamics simulations, and resistance mutation profiles—AI platforms predict high-affinity ligands that disrupt Tat’s functional interfaces while minimizing off-target effects [70]. For instance, generative adversarial networks (GANs) have identified compounds binding the arginine-rich motif (ARM) of Tat (residues 49–57) with sub-nanomolar affinity (Kd = 0.8 nM), effectively blocking its interaction with TAR RNA and Cyclin T1 [76].

A standout example is Q308, a dual-action inhibitor discovered through reinforcement learning-guided virtual screening [70]. This compound not only occupies Tat’s basic domain to sterically hinder P-TEFb recruitment but also recruits the E3 ubiquitin ligase cereblon, tagging Tat for proteasomal degradation. In primary CD4^+^ T cells, Q308 reduces Tat half-life from 4 h to <30 min, selectively inducing apoptosis in infected cells via mitochondrial depolarization (ΔΨm loss > 80%) while sparing uninfected counterparts. AI models further predict resistance-conferring mutations (e.g., LTR Δbulge and Tat R57Q) by analyzing >10,000 HIV-1 sequences, enabling the iterative design of resilient scaffolds like Q308v2, which retains efficacy against 94% of circulating Tat variants.

However, challenges persist. While in vitro studies show >95% suppression of viral transcription, tissue-specific delivery barriers limit in vivo translation. Only 5–10% of systemically administered inhibitors reach CNS reservoirs due to P-glycoprotein efflux and lysosomal trapping in macrophages. Emerging solutions include DNA-encoded nanoparticle libraries (DEL-NPs) that couple AI-predicted inhibitors with blood–brain barrier-penetrant (BBB-penetrant) peptides (e.g., angiopep-2), achieving 15-fold higher brain accumulation in murine models [77]. Additionally, organ-on-chip platforms now validate AI predictions using 3D microphysiological systems of lymphoid tissue, revealing that Tat’s conformational flexibility in nucleolar condensates reduces inhibitor occupancy by 40%—a caveat addressed by dynamic docking algorithms. In addition, although Q308 exhibits high affinity for HIV-1 Tat, its binding to HIV-2 Tat requires further validation. Given the divergence in the ARM sequence and the presence of site-specific chimeric mutants, the binding efficacy is expected to be diminished. For example, proviruses integrated near the MALAT1 locus produce Tat-MALAT1 fusion proteins, which obscure the binding epitope of Q308 and thereby prevent its degradation.

This AI-driven paradigm shift offers unprecedented speed, with lead optimization timelines compressed from years to months. Yet, its success hinges on resolving data scarcity in underrepresented HIV subtypes and integrating single-cell Tat activity maps to prioritize context-dependent vulnerabilities. As computational power converges with wet-lab automation, the goal is clear: to engineer Tat inhibitors that outpace viral evolution, transforming HIV from a chronic adversary to a curable condition.

#### 3.3.2. CRISPR-Based Precision Tools

The CRISPR revolution, fueled by functional metagenomics discoveries and protein engineering, has birthed a versatile arsenal for combating HIV persistence. Beyond canonical Cas9 nucleases, base editors (e.g., BE4max), prime editors (PE2), and dCas9 regulatory systems now enable surgical interventions across the viral life cycle—from eradicating integrated proviruses to silencing Tat-mediated transcription [78]. These tools exploit CRISPR’s modularity: the guide RNA (gRNA) directs specificity, while the effector domain dictates functional outcomes, whether introducing single-nucleotide mutations, rewriting epigenetic marks, or recruiting transcriptional machinery.

Targeted Reactivation: “Shock” via Transcriptional Synergy. The dCas9-VP64-SEC fusion system epitomizes precision latency reversal. By fusing catalytically dead Cas9 (dCas9) with VP64 transactivation domains and subunits of the SEC, this construct recruits endogenous Tat and P-TEFb to the HIV LTR [79]. In resting CD4^+^ T cells, this tripartite complex achieves 3D chromatin looping between the LTR and host enhancers, increasing Tat expression 12-fold and reactivating 78% of latent proviruses—far surpassing HDAC inhibitors like vorinostat (15–20% efficacy). Crucially, gRNAs targeting conserved LTR regions (e.g., NF-κB/Sp1 sites) prevent escape mutations, a limitation of small-molecule LRAs.

Epigenetic Silencing: “Lock” Through Chromatin Rewiring. Conversely, dCas9-KRAB-DNMT3A enforces deep latency by engineering repressive chromatin landscapes. The KRAB domain recruits KRAB-associated protein 1 (KAP1), which deposits H3K9me3 via SET domain bifurcated 1 (SETDB1), while DNMT3A methylates CpG islands at the LTR. In humanized mice, this approach reduces viral RNA by 99.7% for 6 months post-treatment, outperforming dCA’s transient suppression. Single-cell ATAC-seq reveals that edited proviruses acquire bivalent domains (H3K4me3/H3K27me3), rendering them resistant to reactivation by TNF-α or latency-reversing agents.

The Nullbasic mutant—a cirspr-engineered molecular scissor generated by the CRISPR/Cas9 truncation of Tat’s basic domain (Δ residues 49–57)—exemplifies viral disarmament through synthetic biology. Nullbasic retains Tat’s nuclear localization signal but cannot bind TAR RNA or Cyclin T1, reducing transcriptional elongation by 95%. Simultaneously, it sequesters HIV Rev via its intact activation domain, blocking nuclear export of viral RNA. In macaque models, AAV-delivered Nullbasic suppresses plasma viremia below detection for 18 months—yet faces hurdles: 35% of cells show AAV genome integration, and clonal reservoirs with defective Tat loci (Δexon1) escape silencing.

Despite CRISPR’s precision, anatomical and clonal barriers persist. Only 10–15% of CNS microglia are transduced by intravenous AAV9, while LNPs exhibit limited penetration into gut-associated lymphoid tissue. Innovations like manganese-coated DNA nanoballs enhance delivery efficiency 5-fold in non-human primates by evading serum nucleases. To counter off-target effects, HiFi-Cas9 variants reduce unintended edits from 12% to 0.2%, as validated by GUIDE-seq in primary T cells.

Emerging CRISPR-dCas13a systems directly target HIV RNA genomes for degradation, bypassing proviral integration entirely. When combined with Tat-inducible suicide genes (e.g., HSV-TK), they create synthetic kill switches activated only upon viral reactivation—a safeguard against off-tumor toxicity [80]. As CRISPR toolkits evolve, the dream of a mutation-proof cure inches closer, promising to consign Tat’s duality to therapeutic history.

#### 3.3.3. Nanoparticle Delivery Platforms

The transformative success of lipid nanoparticles (LNPs) in mRNA vaccine delivery has spurred their adaptation to HIV cure strategies, particularly for targeting Tat—the viral transactivator central to viral persistence. LNPs encapsulating codon-optimized *tat* mRNA achieve tunable latency reversal in lymphoid reservoirs, leveraging ionizable lipids like SM-102 derivatives (e.g., ALC-0315) to achieve 95% mRNA encapsulation efficiency and pH-dependent endosomal escape [81]. In humanized mice, these LNPs boost Tat expression 3-fold compared to free mRNA, reactivating 65% of latent proviruses in gut-associated lymphoid tissue (GALT) through IL-15/STAT5 co-signaling. Sustained Tat release over 72 h—contrasting with the 6-h activity window of free mRNA—prevents premature apoptosis of reactivated cells, enhancing the efficacy of “shock and kill” strategies. To penetrate the central nervous system (CNS), LNPs are surface-modified with transferrin receptor (TfR)-targeting single-domain antibodies, hijacking receptor-mediated transcytosis to cross the blood–brain barrier. In macaque models, TfR-LNPs achieve 18% brain biodistribution, reducing cerebrospinal fluid viral RNA by 60% after four weekly doses [82]. To enhance CNS delivery, TfR-LNPs cofunctionalized with apolipoprotein E-mimetic peptides may provide a promising dual-strategy approach. This method could effectively improve CNS delivery by promoting transcytosis via low-density lipoprotein receptor-mediated pathways and transiently opening the BBB. Self-amplifying RNA (saRNA) payloads further sustain Tat levels (>200 copies/cell for 10 days), overcoming epigenetic silencing in microglia, while microRNA-9-responsive designs ensure glial-specific delivery, sparing neurons from neurotoxicity and reducing astrocyte activation by 75% [83].

Despite these advances, LNP systems face translational bottlenecks. Unmodified mRNA degrades rapidly, with 50% integrity loss within two weeks at 4 °C—a challenge mitigated by nucleoside substitutions (1-methylpseudouridine) and lyophilization, extending shelf-life to six months at −20 °C. Scalability issues persist: conventional ethanol injection methods yield only 30% encapsulation consistency, whereas microfluidic mixing achieves 90% uniformity (PDI < 0.1) in 10 L batches. Repeated dosing introduces anti-polyethylene glycol immunity, reducing delivery efficiency by 40% by the third week. Innovations like zwitterionic lipids circumvent polyethylene glycol-related immunogenicity while maintaining 80% transfection efficacy. Meanwhile, payload diversification is expanding LNP utility: CRISPR-Cas9 ribonucleoproteins (RNPs) edited 45% of LTRs in CNS reservoirs without genomic integration risks, while the co-delivery of *tat* mRNA and *ccr5* siRNA reduced viral reseeding by 92% in humanized mice. Theragnostic LNPs doped with gadolinium enable the real-time MRI tracking of Tat expression hotspots, correlating strongly with viral RNA levels (r = 0.89, *p* < 0.001).

Looking ahead, LNP platforms hold the potential to revolutionize HIV cure strategies by leveraging anatomical precision and multiplexed payload delivery. However, challenges persist in achieving an optimal balance between immunogenicity and stability. To address these, AI-driven design tools—such as lipid-Tat interactions predicted by AlphaFold—are expediting the optimization process. As Good Manufacturing Practice (GMP) production scales up, LNPs may soon deliver a decisive blow to HIV’s remaining sanctuaries by integrating Tat reactivation, CRISPR editing, and immune modulation into a single nanoscale package. The integration of these technologies promises not just viral suppression but a definitive end to HIV’s four-decade reign—ushering in an era where functional cures transition from aspiration to reality.

#### 3.3.4. Single-Cell Multiomics-Guided Interventions

The advent of single-cell multiomics has unmasked the profound heterogeneity within HIV reservoirs, revealing Tat expression as a critical determinant of clonal persistence and therapeutic resistance. By integrating single-cell RNA sequencing (scRNA-seq) and assay for transposase-accessible chromatin (scATAC-seq), researchers now map Tat expression mosaicism at unprecedented resolution. Proviruses integrated near immune regulatory loci like *BACH2* or *STAT5B* emerge as Tat-high clones, exhibiting 10- to 20-fold elevated Tat mRNA levels compared to other reservoirs [84]. These clones resist didehydro-cortistatin A (dCA) treatment due to *BACH2*-driven enhancer hijacking—a mechanism where host super-enhancers boost Tat transcription while maintaining viral latency through BRD4-mediated chromatin looping. CRISPR barcoding further exposes their dominance: <10% of clonally expanded populations, often harboring intact proviruses in transcriptionally permissive chromatin states (ATAC-seq peaks at NF-κB/Sp1 sites), account for >80% of rebound viremia post-ART cessation.

To dismantle these resilient clones, precision targeting strategies are being deployed: CAR-T cell prioritization: Chimeric antigen receptors (CARs) engineered to recognize Tat-dependent surface markers (e.g., HIV Env-Tat fusion proteins) selectively eliminate clones driving viral rebound, achieving 70% reservoir reduction in humanized mice [85]. Clonal chemosensitization: Small molecules targeting *BACH2*-Tat enhancer complexes (e.g., BET inhibitor JQ1) resensitize resistant clones to dCA, reducing Tat levels by 90% in lymphoid tissues [86].

However, translational roadblocks persist. Single-cell workflows remain prohibitively expensive, with per-cell costs (~USD 1.50) limiting cohort-scale analyses [87]. Computational challenges are equally daunting: aligning scRNA-seq/scATAC-seq datasets from 100,000+ cells requires 500+ GPU hours, while batch effects introduced by tissue dissociation artifacts confound 30% of Tat expression calls. Emerging solutions include machine learning pipelines (e.g., scVI and SCANPY) that denoise data by modeling transcriptional bursting kinetics, and microfluidic platforms (10x Genomics Xenium) enabling spatially resolved Tat RNA–protein co-mapping in archival tissues.

The future lies in multiomics-guided combinatorial therapies. By coupling CRISPR barcoding with in situ CRISPR inhibition (CRISPRi), researchers now track clonal responses to Tat-targeted agents in real time, identifying synergistic regimens (e.g., dCA + PD-1 blockade) that eradicate 99% of rebound-competent clones. As costs decline and algorithms mature, single-cell multiomics will transition from a research tool to a clinical compass—guiding the precise annihilation of HIV’s most virulent reservoirs and consigning Tat’s heterogeneity to therapeutic oblivion.

#### 3.3.5. Combinatorial Synergies

The path to an HIV functional cure demands combinatorial strategies that exploit Tat’s pivotal role while countering viral adaptability and anatomical evasion. By integrating Tat-targeted agents with immune modulators, gene editors, and epigenetic regulators, researchers are engineering multilayered attacks capable of surmounting reservoir heterogeneity. A prime example combines Tat-Oyi vaccines with PD-1/CTLA-4 checkpoint inhibitors, which synergistically resurrect antiviral immunity. In SHIV-infected macaques, Tat-Oyi primes polyfunctional CD4^+^ T cells (IL-2↑5× and IFN-γ↑3×), while anti-PD-1 antibodies reverse T cell exhaustion (PD-1^+^CD8^+^ T cells ↓45%→12%), collectively achieving 90% reservoir reduction in lymph nodes [88]. This synergy capitalizes on PD-1 blockade-mediated reversal of T cell exhaustion alongside Tat’s dual role: as a decoy antigen, extracellular Tat is neutralized by vaccine-induced antibodies, while intracellular Tat-MHC-I complexes guide CTLs to eliminate reactivated cells. However, CNS toxicity—driven by Tat-induced neuroinflammation—requires mitigation through low-dose intrathecal dexamethasone, reducing astrocyte activation (GFAP^+^ cells ↓40%) without compromising efficacy.

Parallel advances merge CRISPR-Tat editors with broadly neutralizing antibodies (bNAbs) to balance precision reactivation and viral containment. Lipid nanoparticle-delivered dCas9-VP64 systems recruit endogenous Tat and host transcriptional machinery to reactivate 85% of latent proviruses in gut-associated lymphoid tissue, while bNAbs like 10-1074 neutralize 99.9% of free virions within hours, slashing reseeding events by 92%. This approach extends to CNS sanctuaries, where microglia-mediated antibody-dependent phagocytosis clears reactivated cells, reducing CSF viral RNA by 70%. Meanwhile, epigenetic combinations—such as dCA (Tat inhibitor) + EZH2 inhibitors—enforce deep latency through dual mechanisms: dCA blocks Tat-P-TEFb binding (IC50 = 2 nM), while EZH2i erases repressive H3K27me3 marks, silencing NF-κB-driven reactivation [89]. Nanoparticle co-formulations (PLGA-EZH2i/dCA) enhance brain delivery, achieving 50% higher drug concentrations without neurotoxicity, a critical advance given Tat’s neurotoxic isoforms (e.g., C31S).

Despite these breakthroughs, sanctuary sites like the CNS demand cautious innovation. Tat’s capacity to trigger TNF-α storms upon reactivation necessitates blood–brain barrier-sparing strategies, such as BBB-impermeant dCA analogs (dCA-COOH) paired with intrathecal bNAbs. Emerging solutions include Tat-dependent suicide genes (HSV-TK + ganciclovir) that selectively eliminate reactivated microglia and real-time PET monitoring ([18F]FEPPA) to adjust dosing when neuroinflammation exceeds safe thresholds (SUV > 2.5) [80]. Looking ahead, AI-driven synergy mapping is reshaping therapeutic design: neural networks trained on single-cell reservoir profiles now predict optimal triple therapies (e.g., Tat mRNA-LNP + anti-CTLA4 + TLR9 agonists), achieving 99% clearance in silico and 85% ex vivo reduction in humanized mice. By harmonizing viral reactivation, immune resurrection, and precision containment, these combinatorial strategies transform Tat from a viral linchpin into a therapeutic pivot—bridging the gap between suppressive ART and definitive cure.

**Table 4:** The key advances and challenges of emerging technologies.

Technology	Advance	Challenge
AI Inhibitors [70]	Q308’s dual-action mechanism	Limited in vivo validation
CRISPR Tools [66,79,90]	Nullbasic’s specificity	Off-target edits in clones
LNPs [72]	Brain-targeted delivery	Scalability for chronic regimens
Single-Cell Multiomics [91]	Identifies Tat-high clones	High cost/complexity
Combinatorial Therapies	90% reservoir reduction in primates	Neurotoxicity risks

## 4. Conclusions

The HIV-1 Tat protein, a master regulator of viral transcription and latency, embodies a paradoxical duality—stabilizing viral dormancy through epigenetic silencing while driving reactivation via transcriptional hijacking. Its exploitation of host machinery, from P-TEFb/7SK snRNP recruitment to EZH2-mediated chromatin compaction, has positioned Tat as both a formidable adversary and a therapeutic linchpin [89]. However, the resilience of HIV reservoirs—forged by clonal expansion dynamics, tissue-specific adaptations (e.g., neurotoxic Tat-C31S in microglia), and integration site-driven heterogeneity—demands strategies that transcend conventional monotherapies.

Emerging technologies are redefining Tat’s role in cure paradigms. AI-designed inhibitors (Q308) and CRISPR-engineered systems (Nullbasic) exemplify precision targeting, suppressing or reactivating Tat with unprecedented specificity [70]. Nanoparticle platforms, such as LNP-encapsulated Tat mRNA, overcome anatomical barriers, achieving 3-fold higher reactivation in CNS reservoirs while mitigating neurotoxicity through glial-specific delivery. Combinatorial synergies—Tat vaccines with PD-1 blockade and CRISPR editors with bNAbs—demonstrate that coupling latency reversal with immune priming can reduce rebound viremia by >90% in preclinical models. Yet, challenges persist: Tat’s pleiotropic roles in immune evasion and neuroinflammation necessitate spatiotemporal control, while clonal escape via dominant-negative Tat mutants (e.g., K41A) underscores the need for adaptive therapeutic design [66].

However, critical knowledge gaps remain unresolved. Future research must address the following: (i) Tat’show phase separation dynamics in nuclear condensates regulate its bistable functionality; (ii) whether clonal lineage tracing technologies can predict reservoir evolution under therapeutic pressure, and (iii) what safety thresholds prevent neurotoxicity during combinatorial “shock and kill” regimens. The road ahead requires convergence of cutting-edge tools and mechanistic insights. Single-cell multiomics will decode Tat expression mosaicism, enabling the CRISPR-barcoded eradication of replication-competent clones. AI must evolve beyond drug design to predict tissue-specific delivery bottlenecks and resistance trajectories, while combinatorial regimens should integrate in situ immune priming (e.g., TLR9 agonists) with epigenetic lock-and-key strategies. Critically, translational frameworks must confront ethical and safety frontiers—minimizing off-target genome editing, curbing neuroinflammatory cascades, and ensuring equitable access to curative therapies.

In this pivotal era, Tat’s duality is no longer a barrier but a blueprint. By orchestrating advances in molecular virology, synthetic biology, and computational analytics, the scientific community is dismantling HIV’s reservoir architecture. Though challenges loom, the synthesis of mechanistic rigor and technological ingenuity illuminates a path where Tat—the viral maestro of persistence—becomes the cornerstone of HIV eradication.

## Figures and Tables

**Table 1:** Mechanisms of Tat in modulating HIV latency.

Mechanism	Category	Functional Outcome
Concentration-dependent bistability	Absence of Tat	Yielding short abortive transcripts
	Presence of Tat	Producing full-length RNA transcripts
Elongation complex	/	Eliminating transcriptional barriers, facilitating transcriptional elongation
Auxiliary mechanisms	Epigenetic modifications	Recruiting acetyltransferases and USP11, phosphorylating EZH2 to promote transcription
	Protein partnerships	Interacting with Vpr, Nef, Rev, and nucleocapsid to bidirectionally regulate latency
	miRNA crosstalk	Regulating the level of miRNA to bidirectionally modulate latency

**Table 3:** The challenges of Tat Targeted Therapies.

Challenge	Technical Solution	Limitations
Organizational barriers (such as the blood–brain barrier)	Targeted LNPs (transferrin receptor-modified)	Delivery efficiency is limited by particle size
Tat functional pleiotropy (neurotoxicity)	CRISPR-Tat mutant (tat-R5M4) reduces toxicity	May affect transcriptional activation efficiency
Heterogeneity of latent reservoirs	Single-cell multiomics identification of Tat high-expression clones	High cost and complex data analysis
Drug resistance	AI-optimized multi-target inhibitors (Tat + Vif dual targets)	Potential off-target risks
Low delivery efficiency	LNP dose optimization and multiple dosing strategies	Immunogenicity risk in chronic treatment
